# PROBIOTICS AS AN ADJUNCTIVE THERAPY FOR CELIAC DISEASE: SYMPTOM RELIEF AND QUALITY OF LIFE IMPROVEMENT

**DOI:** 10.1590/S0004-2803.24612024-111

**Published:** 2025-07-21

**Authors:** Gabriel Fernandes Alves JESUS, Monique MICHELS, Emily CÓRNEO, Marina P ROSSETTO, Alexandre J FARACO, Ana Paula PESARICO

**Affiliations:** 1 Gabbia Biotecnologia, Itajaí, SC, Brasil.; 2 Universidade do Extremo Sul Catarinense, Criciúma, SC, Brasil.; 3 Universidade Federal do Pampa, Programa de Pós-Graduação em Bioquímica, Uruguaiana, RS, Brasil.

**Keywords:** Gluten-free diet, gut microbiota, Gastrointestinal Symptom Rating Scale, Bristol Stool Form Scale, Dieta sem glúten, microbiota intestinal, Escala de Avaliação de Sintomas Gastrointestinais, Escala de Formas das Fezes de Bristol

## Abstract

**Background:**

Celiac disease is a chronic autoimmune disorder triggered by gluten ingestion in genetically predisposed individuals, leading to intestinal damage. Probiotics have been studied for their potential benefits in modulating gut microbiota and alleviating gastrointestinal symptoms, which may be beneficial in managing celiac disease (CD).

**Objective:**

In this way, this study evaluated the effects of probiotics compared to placebo in individuals with CD over the course of treatment.

**Methods:**

A total of 85 participants, with an average age of 40 years, were randomized into two groups using a computer-generated list: 39 receiving placebo and 46 receiving a probiotic blend of *Bifidobacterium lactis* CCT 7858 and *Lactobacillus rhamnosus* CCT 7863 (1 x 10^9^ CFU/day) over 90 days. Participants received either a daily probiotic capsule or an identical placebo made from maltodextrin, provided by Gabbia Biotecnologia Ltda. The randomization process and group assignments were concealed from both participants and investigators.

**Results:**

Both groups exhibited similar demographic and clinical characteristics, with most participants symptomatic for CD and adhering to a gluten-free diet. The Bristol Stool Form Scale (BSFS) showed a predominance of normal stool forms in both groups, with a higher prevalence of type 4 in the probiotic group. Gastrointestinal Symptom Rating Scale (GSRS) scores improved significantly in the probiotic group compared to placebo. Additionally, the probiotic group showed significant improvements in emotional well-being and gastrointestinal symptoms, leading to a better quality of life, as measured by the CD-specific quality of life (CD-QOL) scores.

**Conclusion:**

These results suggest that probiotics contribute to symptom improvement and enhanced quality of life in CD patients.

## HIGHLIGHTS

• Probiotics improve celiac disease symptoms.

• This study involved 85 participants with celiac disease.

• The probiotic group significantly modulated gastrointestinal symptoms and quality of life.

• The probiotics are beneficial adjunct therapy for individuals with celiac disease.

## RESUMO

**Contexto:**

A doença celíaca (DC) é um distúrbio autoimune crônico desencadeado pela ingestão de glúten em indivíduos geneticamente predispostos, levando a danos intestinais. Os probióticos têm sido estudados por seus potenciais benefícios em modular a microbiota intestinal e aliviar sintomas gastrointestinais, o que pode ser benéfico no manejo da DC.

**Objetivo:**

Este estudo avaliou os efeitos dos probióticos em comparação ao placebo em indivíduos com DC ao longo do tratamento.

**Métodos:**

Um total de 85 participantes, com média de idade de 40 anos, foram randomizados em dois grupos usando uma lista gerada por computador: 39 receberam placebo e 46 receberam uma combinação probiótica de *Bifidobacterium lactis* CCT 7858 e *Lactobacillus rhamnosus* CCT 7863 (1 x 10^9^ UFC/dia) por 90 dias. Os participantes receberam uma cápsula probiótica diária ou um placebo idêntico feito de maltodextrina, fornecido pela *Gabbia Biotecnologia Ltda*. O processo de randomização e a atribuição dos grupos foram ocultados de ambos, participantes e investigadores.

**Resultados:**

Ambos os grupos apresentaram características demográficas e clínicas semelhantes, com a maioria dos participantes sintomáticos para DC e aderindo a uma dieta sem glúten. A Escala de Forma das Fezes de Bristol (BSFS) mostrou uma predominância de formas normais de fezes em ambos os grupos, com uma maior prevalência do tipo 4 no grupo probiótico. As pontuações da Escala de Avaliação de Sintomas Gastrointestinais (GSRS) melhoraram significativamente no grupo probiótico em comparação ao placebo. Além disso, o grupo probiótico mostrou melhorias significativas no bem-estar emocional e nos sintomas gastrointestinais, levando a uma melhor qualidade de vida, conforme medido pelas pontuações de qualidade de vida específicas para DC (CD-QOL).

**Conclusão:**

Esses resultados sugerem que os probióticos contribuem para a melhora dos sintomas e a qualidade de vida em pacientes com DC.

## INTRODUCTION

Celiac disease (CeD) is a common autoimmune disorder that primarily affects the structure and function of the small intestine due to an abnormal immune response to gluten, a protein found in wheat and certain other grains^1^. This condition affects at least 48 to 300 million people worldwide^2^ and is more prevalent in women than in men^3^. CeD often presents with adverse gastrointestinal symptoms along with extraintestinal manifestations such as headaches, fatigue, skin conditions, neurological issues, and psychiatric disorders. The only proven treatment to date is strict adherence to a gluten-free diet. However, patients often find it challenging to fully adhere to this diet, leading to the onset of various disease complications.

Genetic predisposition plays a crucial role in CeD, primarily associated with the human leukocyte antigen (HLA-DQ) system, which is involved in the immune system’s recognition of self and non-self molecules^4^. Additionally, the intestinal microbiota significantly contributes to the clinical manifestation of CeD. The microbiota can modulate the digestion of gluten peptides, generating both toxic and tolerogenic peptides that may influence the acquisition of dietary tolerance to antigens. Furthermore, the microbiota can affect intestinal permeability through the release of zonulin and the expression of tight junctions, promote the maturation of mucosal epithelium, and regulate immune system activity by expressing cytokines and peptides with pro-inflammatory or anti-inflammatory properties^5^ ^-^ ^8^.

In this context, probiotics appear to be an intriguing adjuvant in the dietary management of CeD^9^. When ingested in adequate quantities, these live organisms provide health benefits to the host^10^. A systematic review and meta-analysis demonstrated that certain bacterial species belonging to the genera Lactobacillus and Bifidobacterium have protective properties for epithelial cells against damage caused by gluten^11^.

Probiotics could offer promising benefits in managing CeD through three pivotal mechanisms. Firstly, they break down gluten proteins into smaller peptides, potentially mitigating CeD-related pathogenesis and immune responses^12^. Secondly, probiotics bolster intestinal barrier function, effectively blocking immunogenic peptides from infiltrating the lamina propria and triggering inflammatory cascades^13^ ^,^ ^14^. Lastly, probiotics play a crucial role in rebalancing gut microbiota and modulating immune activity by regulating Toll-like receptor expression and cytokine production.

These multifaceted actions not only reduce intestinal inflammation but also enhance the gut’s resilience against pathogens^15^ ^,^ ^16^. Incorporating probiotics as adjunctive therapy in CeD management holds significant therapeutic promise, addressing inflammation and optimizing gastrointestinal health. The objective of this study is to develop a probiotic blend (*Bifidobacterium lactis* CCT 7858; *Lactobacillus rhamnosus* CCT 7863) specifically designed to alleviate symptoms associated with CeD and to significantly improve patients’ overall quality of life.

## METHODS

This was a prospective, double-blind, randomized placebo-controlled parallel group study approved by the Ethics Committee (5.714.353). The patient recruitment was carried out between 2022 and 2023 at the Gastroenterology Units of Criciuma, Santa Catarina, Brazil, by inviting volunteer adult (age 18 y 65) celiac patients. 

The inclusion criteria were: (a) being between 18 and 65 years of age and (b) having a diagnosis of celiac disease confirmed by a gastroenterologist, a specialist in digestive system disorders and diseases. Individuals were excluded if they: (a) were pregnant; (b) were under 18 years of age; (c) had major gastrointestinal pathologies (e.g., Crohn’s disease or ulcerative colitis); (d) were participating in another clinical trial; (e) had decompensated blood pressure or a history of heart disease, including valvular heart disease or any implantable device; (f) had a history of daily consumption of probiotics, fermented milk, and/or yogurt; (g) had previously demonstrated a reaction, including anaphylaxis, to any substance in the study product; or (h) were unwilling to comply with the study protocol or, in the opinion of the investigators, could compromise the study.

### Study design

A randomization list was computer generated in blocks of 85 patients (ie, 39 receiving placebo and 46 active treatments in random order within the block, software by randomization.com). The active study product consisted of a blend of 2 strains: *Bifidobacterium lactis* CCT 7858 and *Lactobacillus rhamnosus* CCT 7863 in the final concentration of 1 x 10^9^ CFU/day during 90 days. The probiotic was given as a capsule once per day. A placebo was made up with the same excipient base as the active formulation, which is maltodextrin, and was identical in size, weight, and packaging to the active medicine. All study products were provided free of charge by Gabbia Biotecnologia Ltda. (Itajai, Brazil), which monitored the stability of the probiotic formulation throughout the study. Group assignment was concealed from participants and investigators. The randomization list defining the given treatment was kept in a sealed envelope that was not opened unless there was a serious adverse event that was deemed by the principal investigators to be directly associated with the study product. Furthermore, the envelopes were kept sealed until all study-related data had been digitalized and the resulting computer file locked. An independent medical staff member assigned subjects to the 2 schedules. Each patient received the next pack of study products stored in the center following the ascending order of labels. Labeling of study products was performed by an outside partner not taking part in the study.

### Study plan and assessment of patients

The study lasted for 90 days (12 weeks) and consisted of three visits. From week 1 to 12, patients filled-in the questionnaires at 4-week intervals during the visits. The visit 1 (day 0) included evaluation of inclusion and exclusion criteria, clinical and physical assessment, presentation of the research, and signing of the Informed Consent Term, partial delivery of the test product or placebo, and administration of two questionnaires: Quality of Life questionnaires (CD-QOL scale), a 20-item self-report instrument with four factor analytically derived subscales: limitations, dysphoria, health concerns, inadequate treatment and Gastrointestinal Symptom Rating Scale (GSRS), a structured and validated questionnaire widely used in the research of GI diseases, was used to assess the severity of current GI symptoms. Additionally, during the first visit, an explanation was given regarding the importance of the questionnaires, correct completion, and possible gastrointestinal changes in the first days of probiotic use. Visit 2 (day 45) consisted of a clinical consultation with a specialist physician, final delivery of the test product or placebo, and questionnaire administration. Finally, the visit 3 (day 90) involved a clinical consultation with a specialist physician and final administration of questionnaires, marking the end of the study. 

Participants were required to immediately report to the study coordinator any events, medication additions, or changes. Details of ill health events and medica-tion changes were recorded and discussed by telephone and at the final interview. Serious ill health and severe stress, for example, hospitalization, were grounds for withdrawal from the trial and reported as serious adverse events.

### Statistical analysis

All data are expressed as median ± SD with 95% confidence intervals unless differently specified. Given the exploratory nature of the study, the sample size calculation was based on the intent to detect a 25% difference in the proportion of responders between treatment and placebo groups; The Friedman test was used, as appropriate, to compare percentages and nominal variables. For continuous variables, differences between patients in the two treatment arms were compared using analysis of variance, and the Wilcoxon test was used for comparison of the mean values. All statistical tests were two tailed and performed at the 5% level of significance. All analyses were performed on the intention-to-treat basis and per protocol analysis. 

## RESULTS

### Demographics

Eighty-five people were screened in the first visit against the inclusion and exclusion criteria. Regarding demographic data, the overall average age was 40 years, and these participants were divided in two groups: Placebo averaging 38 years and Probiotic averaging 41 years. Concerning the age at diagnosis of CD, the overall average was 32 years, with Placebo at 31 years and Probiotic at 33 years. Females predominated in both groups, and the majority of volunteers had completed higher education. The most prevalent marital status was single, with steady employment and a monthly income of 1 to 3 minimum wages also being more common among the study population (Table 1).

**TABLE 1:** Comparison of demographic indicators.

Demographics	**Média ± DP, *n* (%)**		
	Total	Placebo	Probiotic
	n=85	n=39	n=46
Age mean (± DP)	40.15±11.10	38.74±12.12	41.35±10.14
Age of diagnosis (± DP)	32.81±11.18	31.62±11.69	33.83±10.77
Gender: males	6 (7.1)	1 (2.6)	5 (10.9)
Gender: females	79 (92.9)	38 (97.4)	41 (89.1)
Primary Education	2 (2.4)	2 (5.1)	0 (0.0)
Lower Secondary	2 (2.4)	2 (5.1)	0 (0.0)
Incomplete Secondary Education	1 (1.2)	0 (0.0)	1 (2.2)
Complete Secondary Education	17 (20.0)	5 (12.8)	12 (26.1)
Incomplete Higher Education	11 (12.9)	6 (15.4)	5 (10.9)
Complete Higher Education	35 (41.2)	17 (43.6)	18 (39.1)
Postgraduate Education	17 (20.0)	7 (17.9)	10 (21.7)
Married	24 (63.5)	14 (35.9)	10 (21.7)
Single	54 (28.2)	22 (56.4)	32 (69.6)
Divorced	6 (7.1)	2 (5.1)	4 (8.7)
Widowed	1 (1.2)	1 (2.6)	0 (0.0)

### Participant’s clinical characteristics

All participants met the diagnostic criteria/definition of CD outlined by the specialist. The majority of volunteers were symptomatic for CD (placebo: 31 volunteers; probiotics: 35 volunteers). Some participants adhered to a gluten-free diet (placebo: 33 volunteers; probiotics: 34 volunteers) and some participants were classified as eutrophic based on the body mass index (BMI) (placebo: 25 volunteers; probiotics: 28 volunteers). 

Despite some volunteers having comorbidities and using antidepressants, most of the participants did not have other diseases (placebo: 24 volunteers; probiotics: 31 volunteers) and did not use the specific medication class (placebo: 29 volunteers; probiotics: 33 volunteers) (Table 2).

**TABLE 2:** Participant’s clinical characteristics.

	***n* (%)**		
	Total	Placebo	Probiotic
	n=85	n=39	n=46
Syntomatic			
Yes	66 (77.6)	31 (79.5)	35 (76.1)
No	19 (22.4)	8 (20.5)	11 (23.9)
Adherence to the gluten-free diet			
Always	67 (78.8)	33 (84.6)	34 (73.9)
Almost always	4 (4.7)	2 (5.1)	2 (4.3)
Sometimes	12 (14.1)	4 (10.3)	8 (17.4)
Almost never	1 (1.2)	0 (0.0)	1 (2.2)
Never	1 (1.2)	0 (0.0)	1 (2.2)
Comorbidities			
Yes	30 (35.3)	15 (38.5)	15 (32.6)
No	55 (64.7)	24 (61.5)	31 (67.4)
Antidepressants			
Yes	23 (27.1)	10 (25.6)	13 (28.3)
No	62 (72.9)	29 (74.4)	33 (71.7)
Body mass index			
Underweight	1 (1.2)	1 (2.6)	0 (0.0)
Normal weight	53 (62.4)	25 (64.1)	28 (60.9)
Overweight	22 (25.9)	11 (28.2)	11 (23.9)
Obesity class I	8 (9.4)	2 (5.1)	6 (13.0)
Obesity class II	1 (1.2)	0 (0.0)	1 (2.2)

### Bristol stool form scale

From the beginning to the end of the treatment, the predominant stool type in the placebo group was type 3. In the probiotic group, type 4 was the most common among the volunteers (Table 3). It is understood that both groups showed a predominance of types 3 and 4, which are categorized as normal stool forms. However, it is important to note that the probiotic group had a higher number of individuals exhibiting type 4 stool throughout the study. After treatment, BSFS scores did not differ in patients receiving probiotics or placebo as absolute values.

**TABLE 3:** Bristol Stool Form Scale (BSFS).

BSFS	***n* (%)**					
	Days					
	0		45		90	
	Placebo	Probiotic	Placebo	Probiotic	Placebo	Probiotic
	n=29	n=38	n=29	n=38	n=29	n=38
Type 1	4 (13.8)	7 (18.4)	1 (3.4)	1 (2.6)	4 (13.8)	1 (2.6)
Type 2	4 (13.8)	9 (23.7)	8 (27.6)	6 (15.8)	4 (13.8)	5 (13.2)
Type 3	7 (24.1)	6 (15.8)	9 (31.0)	11 (28.9)	8 (27.6)	9 (23.7)
Type 4	5 (17.2)	9 (23.7)	6 (20.7)	17 (44.7)	5 (17.2)	22 (57.9)
Type 5	5 (17.2)	2 (5.3)	4 (13.8)	2 (5.3)	4 (13.8)	1 (2.6)
Type 6	3 (10.3)	3 (7.9)	1 (3.4)	1 (2.6)	4 (13.8)	0 (0.0)
Type 7	1 (3.4)	2 (5.3)	0 (0.0)	0 (0.0)	0 (0.0)	0 (0.0)

### Gastrointestinal symptom rating scale (GSRS)

The GSRS was at baseline 2.99 in the probiotic and 3.21 in the placebo group; at the end of treatment, GSRS changed to 1.58 and 2.4, respectively (Table 4). At the end of follow-up, when expressed as variation over the pretreatment value, GSRS in probiotics was significantly decreased, as compared with placebo (*P*<0.001).

**TABLE 4:** Gastrointestinal Symptom Rating Scale (GSRS).

GSRS		Média ± DP			**Valor-*P***
		Days			
	n	0	45	90	
Placebo	39	3.21±1.06	2.35±1.07	2.46±0.88	<0.001
Probiotic	46	2.99±0.90	1.94±0.53	1.58±0.57	

### CD-specific quality of life

Analyzing the data, it was noted that comparing the beginning to the end of the treatment, probiotic group showed a significant improvement in emotional issues, concerns, and gastrointestinal levels compared to placebo group, proving that probiotic group demonstrated a better quality of life at the end of the study (Table 5). After treatment, CD-QOL scores differ in patients receiving probiotics or placebo values (94 and 121, respectively; *P*=0.001). 

**TABLE 5:** Celiac Disease Quality of Life Survey (CD-QOL).

	Média ± DP				
	Days				
CD-QOL	0		90		**Valor-*P*** ^¥^
	Placebo	Probiotic	Placebo	Probiotic	
	n=37	n = 40	n = 37	n = 40	
Emotional	27.03 ± 9.06	30.98±10.34	20.76±15.32	31.80±17.11	0.001
Social	36.95 ± 6.25	39.48±6.05	27.08±17.60	33.40±17.57	0.370
Concerns	26.57 ± 7.22	26.30±7.70	19.62±13.81	21.13±12.85	0.001
Gastrointestinal	35.35 ± 6.76	35.13±7.82	26.54±17.42	35.58±18.35	0.001
Total	125.89 ± 21.17	131.88±20.17	94.00±61.14	121.90±62.72	0.001

## DISCUSSION

In this study, we observed that the group receiving probiotic treatment showed significant improvement in gastrointestinal symptoms, including abdominal distension, flatulence, diarrhea, and constipation. Additionally, there was a marked enhancement in their quality of life, particularly in emotional well-being, concerns, and gastrointestinal health. These findings confirm the safety of the probiotic blend, as evidenced by the positive outcomes obtained.

The demographic characteristics provide a crucial context for interpreting the study results, influencing both adherence to treatment and perception of its effects. Equally important are the clinical characteristics of the participants, all of whom met the diagnostic criteria for CeD, with the majority presenting symptoms and adhering to a gluten-free diet. Many participants were classified as eutrophic. Although some volunteers had comorbidities and were using antidepressants, most did not have other diseases or use this specific class of medication. These clinical data are essential for understanding the overall health condition of the participants and the potential influence of additional factors on the study’s outcome.

As mentioned, many participants follow a gluten-free diet, the primary therapy for CeD, requiring the complete exclusion of dietary sources of grains and any food containing gluten. However, several other treatments have been identified, including genetically modified gluten, zonulin inhibitors, therapeutic vaccines, tissue transglutaminase inhibitors, and, more recently, probiotics^17^. Some studies have consistently reported abnormalities in the intestinal microbiota of CeD patients compared to healthy controls, regardless of adherence to a gluten-free diet. These abnormalities are mainly characterized by an increase in gram-negative bacteria (Bacteroides and enterobacteria) and a decrease in gram-positive bacteria, such as bifidobacteria^18^ ^-^ ^21^. Thus, supplementation with probiotics could be an important and valuable asset in enhancing the quality of life for these patients.

Probiotics have demonstrated the capacity to modulate gut microbiota in both animal models and human clinical trials^22^ ^,^ ^23^. For example, Bifidobacterium, a predominant beneficial genus in the human intestines, is crucial for maintaining gut homeostasis. Dysbiosis, characterized by alterations in the abundance and composition of gut microbiota, is a common feature of various pathologies^24^. A study has shown the ability of probiotics to hydrolyze immunogenic gluten peptides, thus reducing their immunogenicity^25^.

The 15-item GSRS, a structured and validated questionnaire widely used in gastrointestinal research, was employed to assess the severity of current GI symptoms. Notably, the GSRS scores significantly decreased in the probiotic group, even though the baseline scores were higher for participants receiving the probiotic compared to those on placebo. This finding further supports the efficacy of the probiotic combination. We hypothesize that this effect may be partially due to the positive modification of gut microbiota by the probiotic, as previous studies have demonstrated a steady and persistent increase in bifidobacteria counts in fecal samples from CeD patients^26^ ^,^ ^27^. 

Additionally, we observed that the probiotic combination, administered daily for 90 consecutive days, was superior to the placebo in improving the CD-QoL scores, encompassing emotional, social, and gastrointestinal symptoms. Most participants in this study adhered to a gluten-free diet. These results are consistent with some studies suggesting that improvements in quality of life are associated with dietary adherence but inconsistent with others reporting no differences in QoL scores between patients with full adherence and those with partial or non-adherence to a gluten-free diet^26^ ^,^ ^28^ ^,^ ^29^.

No clinically significant improvement in stool appearance was observed between the treatment groups, potentially due to the intricate nature of the disease. This underscores the hypothesis that patients’ perceived health status is multifaceted, influenced not only by symptoms but also by the complex interactions of dietary restrictions with various factors, particularly social dynamics. Another study limitation is the absence of intestinal biopsies to explore the association between symptom persistence and markers of mucosal inflammation.

## CONCLUSION

The group receiving the probiotic treatment exhibited significant improvements in gastrointestinal symptoms, including abdominal bloating, flatulence, diarrhea, and/or constipation. Additionally, enhancements in quality of life were observed, particularly in emotional well-being, reduced concerns, and gastrointestinal health. These findings not only demonstrate the efficacy of the probiotic blend but also confirm its safety. The positive outcomes suggest that this probiotic blend could be a valuable therapeutic option for managing symptoms and improving the overall quality of life in patients with gastrointestinal disorders. Further research is warranted to explore the long-term benefits and mechanisms underlying these effects.
